# Revealing immune infiltrate characteristics and potential immune-related genes in hepatic fibrosis: based on bioinformatics, transcriptomics and q-PCR experiments

**DOI:** 10.3389/fimmu.2023.1133543

**Published:** 2023-04-14

**Authors:** Yan-Ming Bai, Shuang Liang, Bo Zhou

**Affiliations:** ^1^ School of Traditional Chinese Medicine, Ningxia Medical University, Yinchuan, China; ^2^ Yinchuan Hospital of Traditional Chinese Medicine, Ningxia Medical University, Yinchuan, China; ^3^ Ningxia Regional Key Laboratory of Integrated Traditional Chinese and Western Medicine for Prevention and Treatment of High Incidence, Ningxia Medical University, Yinchuan, China

**Keywords:** hepatic fibrosis (HF), inflammatory, hepatic fibrosis immune genes (HFIGs), hub genes, transcriptomics

## Abstract

**Background:**

The occurrence and progression of hepatic fibrosis (HF) is accompanied by inflammatory damage. Immune genes play a pivotal role in fibrogenesis and inflammatory damage in HF by regulating immune cell infiltration. However, the immune mechanisms of HF are inadequately studied. Therefore, this research aims to identify the immune genes and biological pathway which involved in fibrosis formation and inflammatory damage in HF and explore immune target-based therapeutics for HF.

**Methods:**

The expression dataset GSE84044 of HF was downloaded from the GEO database. The crucial module genes for HF were screened according to weighted gene co-expression network analysis (WGCNA). The crucial module genes were mapped to immune-related genes obtained from the ImmPort database to obtain the hepatic fibrosis immune genes (HFIGs). In addition, Gene Ontology (GO) and Kyoto Encyclopedia of Genes and Genomes (KEGG) functional enrichment analyses were performed on HFIGs. Then, the protein-protein interaction (PPI) network was conducted on HFIGs and hub genes were identified from the PPI network. Moreover, immune infiltration analysis was performed to identified correlation between hub gene and immune cell infiltration. To verify the reliability of the GSE84044 expression profile data analysis, a rat model of CCl4-induced HF was established, followed by transcriptome sequencing and immunofluorescence analysis and quantitative reverse transcription (q-PCR) experiments were performed in HF rats and normal rat liver tissues. Finally, CMAP platform was used to explore immune target-based therapeutics for HF.

**Results:**

In the bioinformatics analysis of GSE84044 data, 98 HFIGs were screened. These genes were mainly involved in inflammation-related biological pathways such as NOD-like receptor signaling pathway, NF-kappa B signaling pathway, Toll-like receptor signaling pathway and PI3K-Akt signaling pathway. From the PPI network, 10 hub genes were identified, including CXCL8, IL18, CXCL10, CD8A, IL7, PTPRC, CCL5, IL7R, CXCL9 and CCL2. Immune infiltration analysis showed that immune cells like neutrophils, natural killer (NK) cells, macrophages M1 and macrophages M2 were significantly correlated with the hepatic fibrosis process and hub gene expression was significantly correlated with these immune cells. Notably, most of the biological pathways HFIGs riched and all the hub gene expression except CXCL8 were validated in subsequent transcriptome and qRCR experiments. Finally, 15 small molecule compounds with the potential to reverse the high expression of hub genes were screen out as potential therapeutic agents for HF.

**Conclusion:**

The immune genes CXCL8, IL18, CXCL10, CD8A, IL7, PTPRC, CCL5, IL7R, CXCL9 and CCL2 may play an essential role in the fibrosis formation and inflammatory damage in HF. The outcomes of this research provide a basis for the study of the immune mechanisms of HF and contribute to the diagnosis and prevention and treatment of HF in clinical practice.

## 1 Introduction

Hepatic fibrosis (HF) is an excessive repair response caused by various chronic liver diseases such as viral hepatitis, alcoholic hepatitis, cholestasis, and drug-induced liver injury (1). HF is an increasing public health concern worldwide, and may lead to the occurrence of serious high-risk liver diseases such as cirrhosis and liver cancer (2, 3). However, there are no highly effective drugs for the treatment of HF approved for clinical use (4). Therefore, exploring the pathogenesis of HF has an important significance for the discovery of targeted anti-hepatic fibrosis drugs.

HF occurs directly due to the excessive production and deposition of extracellular matrix (ECM) including collagen, glycoproteins and proteoglycans (5). The occurrence of HF involves multiple cellular changes. The hepatic stellate cells (HSCs) activation and conversion to myofibroblasts due to various pathogenic factors are central to the HF development. Activated HSCs induce the formation of HF by producing large amounts of ECM (1).

The process of HF is always accompanied by a liver inflammatory damage. Liver inflammation and the hepatic immune microenvironment changes are considered to be key factors in the development of HF (6). The liver has an abundance of immune cells, including innate immune cells (Kupffer cells, natural killer cells, natural killer T cells) and acquired immune cells (T cells and B cells) (7). An increasing number of studies have shown that immune cells regulate the progression and regression of HF. In the process of HF, the immune system is involved in wound healing and tissue repair by triggering inflammation (6). After liver injury, immune cells in the liver are activated and recruited to the site of injury to activate HSCs or injured hepatocytes by secreting pro-inflammatory cytokines such as tumor necrosis factor α (TNF-α), interleukin-6 (IL-6), interleukin-1β (IL-1β), etc (6, 8). Immune related genes and signaling pathways play an important role in the immune infiltration of liver tissue in HF. For example, Interleukin-17 (IL-17), an effector molecule of CD4+ T (Th17) cells, activates macrophages (Kupffer cells) in the liver, which further promotes hepatic stellate cell activation and induces hepatocyte death by producing inflammatory cytokines (9).

In recent years, with the development of sequencing technology, bioinformatics has become a good analytical method widely used for the identification of biomarkers, pathological mechanisms and potential therapeutic drugs for diseases. For example, Z. Wang et al. (10) used weighted correlation network analysis (WGCNA) to screen several viable diagnostic biomarkers for depression from the peripheral blood of patients with depression. This study integrated bioinformatics, transcriptomics, and animal experiments to identify immune genes and biological pathways associated with HF. This study will help us to further understand the pathogenesis of HF and contribute to its diagnosis and treatment.

## 2 Materials and methods

### 2.1 Microarray data

The GSE profiles (GSE84044) based on GPL570 (Affymetrix Human Genome U133 Plus 2.0 Array) was selected and downloaded from the GEO database (https://www.ncbi.nlm.nih.gov/geoprofiles/). GSE84044 dataset was established and evaluated by Wang M et al. (11). Unlike their focus on fibrosis-related genes, the present study would focus on the immune mechanisms in the development of HF. GSE84044 dataset contained a gene expression matrix and clinical information of 124 patients with HF. Clinical information for each HF patient included gender, age, grading of inflammation (Scheuer score ‘G’) and Histological stage of fibrosis (Scheuer score ‘S’). Scheuer score was used to evaluate liver inflammation and fibrosis. G0-G1, G2 and G3-4 were defined as no or mild, moderate and severe liver inflammation respectively; S0-S1, S2-S3 and S4 indicated no significant hepatic fibrosis, moderate hepatic fibrosis, severe hepatic fibrosis or cirrhosis, respectively. After standardization of GSE84044 gene expression matrix, the gene expression matrix and clinical information of GSE84044 were used for the construction of a gene co-expression network.

### 2.2 Weighted Gene Co-Expression Network Analysis (WGCNA)

The Weighted Gene Co-Expression Network Analysis (WGCNA) of GSE84044 gene expression matrix was performed using R software (version: 3.6.3). Briefly, the top 50% of genes with the smallest MAD (Median Absolute Deviation) were eliminated and the outlier genes and samples were also removed. A weighted adjacency matrix was constructed. Then, the appropriate soft-thresholding value β was chosen and a topological overlap matrix (TOM) was transformed from the adjacency matrix. The average linkage hierarchical clustering and dynamic tree cut algorithm were used to identify gene co-expression modules. To obtain modules related to clinical features, module eigengenes (MEs), as the first principal component of the module, were used to compute the correlation with clinical traits and the modules with pvalue <  0.001 were identified to be clinical traits associated modules. In the study, Common significant modules related to fibrosis and inflammation were screened. Finally, gene significance (GS) and module membership (MM) were calculated, the module genes with GS > 0.4 and MM > 0.5 and pvalue < 0.05 were identified as crucial module genes and the common crucial module genes related to fibrosis and inflammation were identified for subsequent analysis.

### 2.3 Hepatic fibrosis immune genes (HFIGs) identification

2483 immune-related genes (IRGs) were obtained from the ImmPort database (https://www.immport.org/shared/home). The overlapping genes between IRGs and crucial module genes related to fibrosis and inflammation were screened out and identified as hepatic fibrosis immune genes (HFIGs).

### 2.4 Function enrichment analysis of hepatic fibrosis immune genes (HFIGs)

DAVID Database (https://david.ncifcrf.gov/) is a reliable program involved in functions like functional annotation, gene functional classification, gene ID conversion. In this study, DAVID Database was used to performed Gene Ontology (GO) and Kyoto Encyclopedia of Genes and Genomes (KEGG) analyses of HFIGs. The enrichment terms with pvalue < 0.05 were considered as significant enrichment results.

### 2.5 The PPI network construction and hub genes screening

The 98 HFIGs were uploaded to the String database (https://string-db.org/) for protein-protein interaction (PPI) analysis. PPI networks were constructed using Cytoscape software. Then, 10 hub genes were identified from the PPI network using the CytoHubba plugin based on the Maximal Clique Centrality (MCC) algorithm. To further explore the biological functions of hub genes, 10 hub genes were uploaded to the Metascape database (http://metascape.org/gp/index.html#/main/step1) for functional enrichment analysis.

### 2.6 Immune infiltration analysis

The role of the immune microenvironment in the formation of HF was analyzed using the CIBERSORTx database (https://cibersortx.stanford.edu/). Gene expression data from each patient in the GSE84044 dataset were uploaded to the CIBERSORTx database to calculate the level of infiltration of 22 immune cells, and then correlations between immune cells and clinical trait score (fibrosis stage and inflammatory grade) and between hub genes and immune cells were analyzed.

### 2.7 Experimental animals and grouping

Male Sprague-Dawley rats (180–220 g) were provided by the Laboratory Animal Center of Ningxia Medical University. The animal experiment was approved by the Ethical Committee of Ningxia Medical University (IACUC-NYLAC-2021-009). Rats were maintained on a 12 h light/dark cycle under a favorable environment (22–24 °C, 45–50% humidity) and had free access to water and rodent chow.

After 1 week of acclimatization, the animals were randomly divided into the normal control and CCl4-induced (Bodi Chemical, Tianjin, China) model group of 6 animals each. The rats in CCl4-induced model group received olive oil with CCl4 subcutaneously at a dose of 23 mL/kg twice a week for 6 weeks to induce hepatic fibrosis, and olive oil was given to normal control rats, and the rats in the normal control simultaneously were received the same volumes of olive oil. 24 hours after the last subcutaneous injection, all rats were anesthetized with 3% pentobarbital sodium (Bodi Chemical, Tianjin, China). The liver tissues were taken for further examination including histological analysis, ELISA assay, transcriptome sequencing and q-PCR assay.

The study protocol involving the use of experimental rats was reviewed and approved by the Research Ethics Committee of Ningxia Medical University and was in accordance with the Animal Management Rules of the Ministry of Health of the People’s Republic of China.

### 2.8 Histological analysis

The liver tissues were fixed in 10% formalin for 24 h, dehydrated, embedded in paraffin and then cut into 5 mm tissue sections. Liver sections were stained with hematoxylin and eosin (HE) (Solarbio, Beijing, China) staining for histological examination and stained with Masson’s Trichrome (Sangon, Shanghai, China) staining to assess the degree of hepatic fibrosis.

### 2.9 Estimation of hepatic fibrosis and inflammation parameters

The liver collagenic parameters markers Hyaluronic acid (HA) and Laminin (LN) were measured by ELISA kit (Bioswamp, Wuhan, China) according to the manufacturer’s protocol. The levels of interleukin-1β (IL-1β), interleukin-6 (IL-6) and tumor necrosis factor α (TNF-α) in liver tissues were estimated by using ELISA kit (Lc-Bio Technologies, Hangzhou, China) according to the manufacturer’s instructions.

### 2.10 Immunofluorescence staining

Here, immunofluorescence staining was performed to detect the infiltration of each immune cell. The expression of Cd10 (Abcam, Cambridge, UK), Cd11b (Abcam, Cambridge, UK), Cd86 (Abcam, Cambridge, UK) and Cd206 (Abcam, Cambridge, UK) were detected to assess the infiltration status of neutrophils, natural killer (NK) cells, macrophages M1 and macrophages M2, respectively. Briefly, After dewaxing to water and antigen repair, the paraffin-embedded liver tissue sections were incubated in 3% hydrogen peroxide for 10 minutes to quench endogenous peroxidase activity. The sections were then permeabilized with 0.2% Triton X-100 (Abcam, Cambridge, UK) for 15 min and blocked with 5% BSA (Abcam, Cambridge, UK) for 30 min. Tissue slices were incubated primary antibodies at 4°C overnight and then were treated with secondary antibodies at room temperature for 30 min. Nuclei were counterstained with 4,6-diamidino-2-phenylindole (DAPI) (Thermo Fisher Scientific, Wilmington, DE) and the slices were mounted in Pro-Long Diamond Antifade Mountant (Thermo Fisher Scientific, Wilmington, DE). Sections were observed and captured with a fluorescence microscope (Nikon Corporation, Tokyoratio.

### 2.11 RNA extraction and cDNA library generation

Total RNA was extracted from liver tissue of 6 rats (3 each for the Model and control groups) using TRlzol Reagent (Life technologies, California, USA) according to the manufacturer’s instructions. The integrity of total RNA was determined using Agilent Bioanalyzer 2100 system (Agilent Technologies, CA, USA) and the concentration and purity of total RNA were detected by NanoDrop 2000 (Thermo Fisher Scientific, Wilmington, DE). The cDNA library was generated using NEBNextUltra™ RNA library prep kit for Illumina (NEB, Ipswich, MA, USA) following manufacturer’s recommendations and the index codes were added to attribute sequences to each sample. The library quality was developed on Agilent Bioanalyzer 2100 system (Agilent Technologies, CA, USA). After cluster generation which was performed on a cBot Cluster Generation System using TruSeq PE Cluster Kit v4-cBot-HS (Illumia) according to the manufacturer’s instructions, the library were sequenced on an Illumina platform.

### 2.12 RNA-seq data processing and analysis

Clean reads were obtained by removing reads containing adaptor sequences, adaptor sequences and reads containing ploy-N from raw data. Quantification of gene expression levels was then calculated using the reads per kilo bases per million reads (RPKM) method. | Fold change |≥1.5 and adjusted pvalue<0.01 were defined as the threshold for screening differentially expressed genes (DEGs) using the DESeq2. The expression of 10 hub genes was compared in normal control and model groups, and the expression correlations between hub genes were calculated.

To verify the reliability of the functional enrichment analysis results of HFIGs, the DEGs of normal rats and hepatic fibrosis rats in RNA-seq data were used for KEGG function enrichment analysis by DAVID database and GSEA analysis respectively. At first, DEGs were uploaded to the DAVID database for KEGG pathway enrichment analysis. pvalues were calculated using the Benjamini-corrected modified Fisher’s exact test and pvalue<0.05 was defined as a threshold of significance. Then, Gene Set Enrichment Analysis (GSEA) was performed on the transcriptome expression matrix using GSEA v4.0.3 software, the KEGG pathways with |NES|>1 (Normalized enrichment score) and pvalue < 0.05 were considered to be significantly enriched.

### 2.13 Quantitative reverse transcription-PCR (q-PCR)

Total RNA of each sample was extracted using Trizol. The total RNA was reverse‐transcribed into cDNA using the PrimeScript RT Master Mix kit (Takara Bio, Kusatsu, Japan). The mRNA expression of hub genes was quantitated by q-PCR using SYBR Green PCR Mix (Monad Biotech, Wuhan, China) on an CFX Connect machine (Applied BIO-RAD, Hercules, CA, USA). β-actin was used as internal control. Primers used in q‐PCR are listed in **Table 1**.

**Table 1:** Primer sequences for q-PCR assay.

Gene	Forward primer (5′–3′)	Reverse primer (5′–3′)	Product size (bp)
Il18	GCGGAGCATAAATGACCAAGTT	GGTCTGGGATTCGTTGGCTGTT	100
Cxcl10	TGCACCTGCATCGACTTCCA	TCTTTGGCTCACCGCTTTCA	189
Cd8a	CTTCAGTCCTCTGGTGCCG	CCCAATCCCATTCCCTCC	165
IL7	TAAAGACAAGGACGGTAAAGC	TTTGGGCAATCACTATCAGTT	93
Ccl5	GCATCCCTCACCGTCATCCTC	GCACTTGCTGCTGGTGTAAAA	156
Il7r	GAAGCAGGGACATAGAGCAAC	TTCTAGCCAGGCATCTTAGGG	110
Cxcl9	ATGAAGTCCGTTGCTCTATT	AGGTCTTTGAGGGATTTGT	143
Ccl2	CAGGTGTCCCAAAGAAGCTGTA	CTGAAGTCCTTAGGGTTGATGC	167
Ptprc	AGATGAACAGCAGGAACTCG	CAGCCAGGAACAGTCTACCC	131
β-actin	AGATTACTGCCCTGGCTCCTAG	CATCGTACTCCTGCTTGCTGAT	144

### 2.14 Identification of candidate small molecules

Connectivity Map (CMAP) database (https://clue.io) is a reliable platform for predicting potential small molecules that may reverse or induce the expression of genes encoded in specific biological states. The 10 hub genes upregulated in liver fibrosis were introduced into the CMAP database to screen for small molecules with potential therapeutic effects in HF. The results were ranked by connectivity score. Small molecules with negative connectivity scores indicating the ability to reverse the upregulation of hub gene expression in HF, were considered to have anti-hepatic fibrosis potential.

### 2.15 Plotting and statistical analysis

The “ggplot2” software package is used for image generation in bioinformatics analysis. GraphPad Prism 8.3.0 software (San Diego, CA, USA) was used for statistical analysis and image construction. Pearson correlation was used to evaluate the linear relationship between data. The experimental data were presented as mean ± standard deviation (SD). Comparisons between groups was evaluated using the Student’s t-test. *pvalue< 0.05, **pvalue< 0.01, ***pvalue< 0.001 were considered significant.

## 3 Results

### 3.1 WGCNA analysis and HFIGs identification

**Figure 1:**
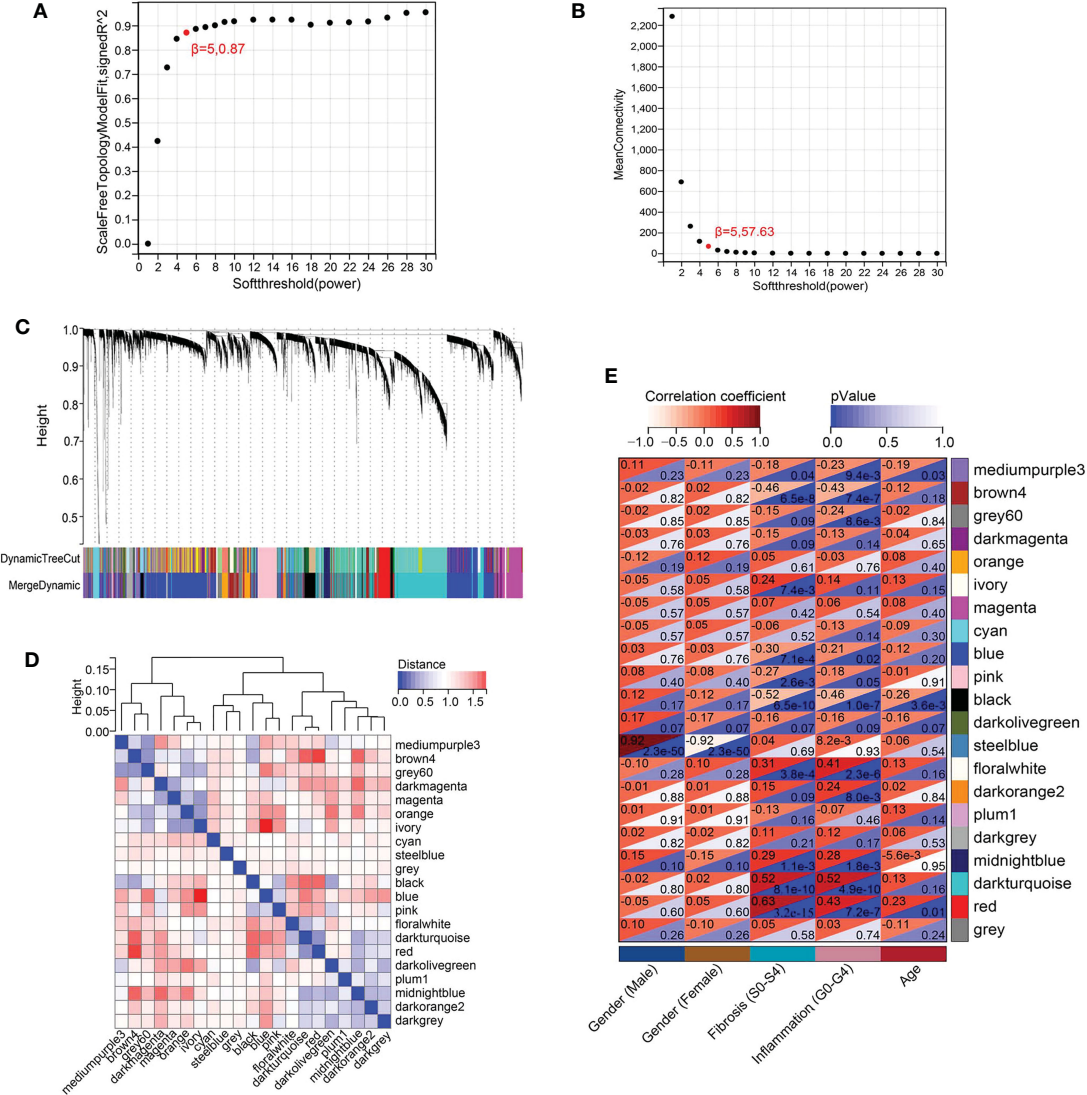
WGCNA of GSE84044 data. **(A)** Scale independence as a function of soft threshold power. **(B)** Mean connectivity as a function of soft threshold power. **(C)** Cluster dendrogram. Each color represents a specific co-expression module. The two-colored rows below the cluster tree represent the original module and the merged module. **(D)** Eigengene dendrogram and eigengene adjacency of module eigengenes (MEs). **(E)** Heatmap of the correlation between MEs and clinical traits.

After standardization, The WGCNA analysis of GSE84044 data was performed. To ensure the network was scale-free and more biological significance, the optimal soft threshold β was set as 5 (**Figures 1A, B**). Then, based on the DynamicTreeCut algorithm, setting the minimum number of module gene as 30, the sensitivity dynamic tree cut (deep split) as 3, and the maximum module distance as 0.25, the gene modules were generated and the modules with high similarity were further merged. As showed in **Figure 1C**, 21 gene modules were finally generated after merging. Eigengene dendrogram and eigengene adjacency were ploted to analyze the connectivity of module eigengenes (MEs), and the results showed that the distance between modules was greater than 0.25 (**Figure 1D**).

**Figure 2:**
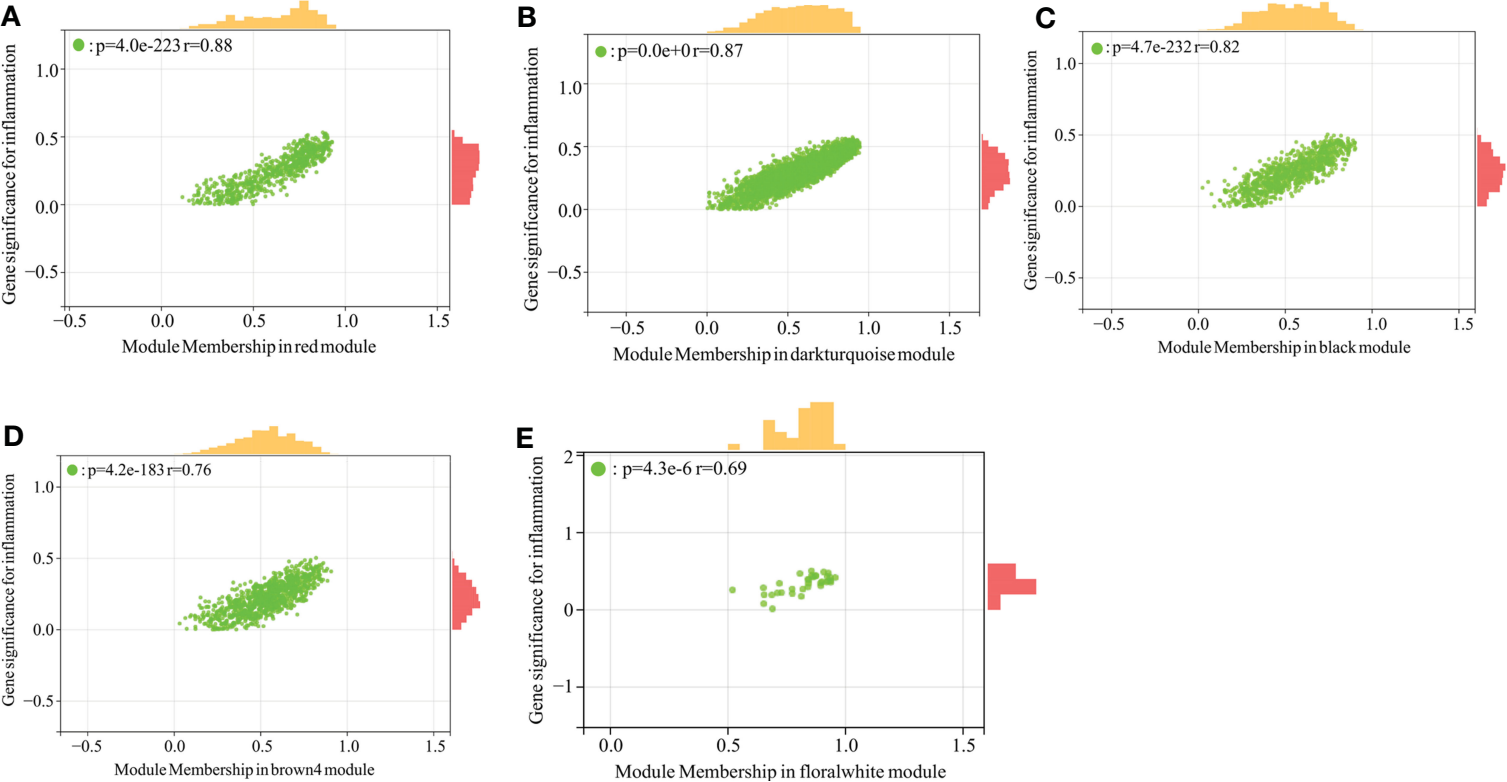
Scatter plot analysis of the modules associated with inflammation trait. **(A–E)**: Scatter plot analysis of the module of red, darkturquoise, black, brown4 and floralwhite, respectively.

**Figure 3:**
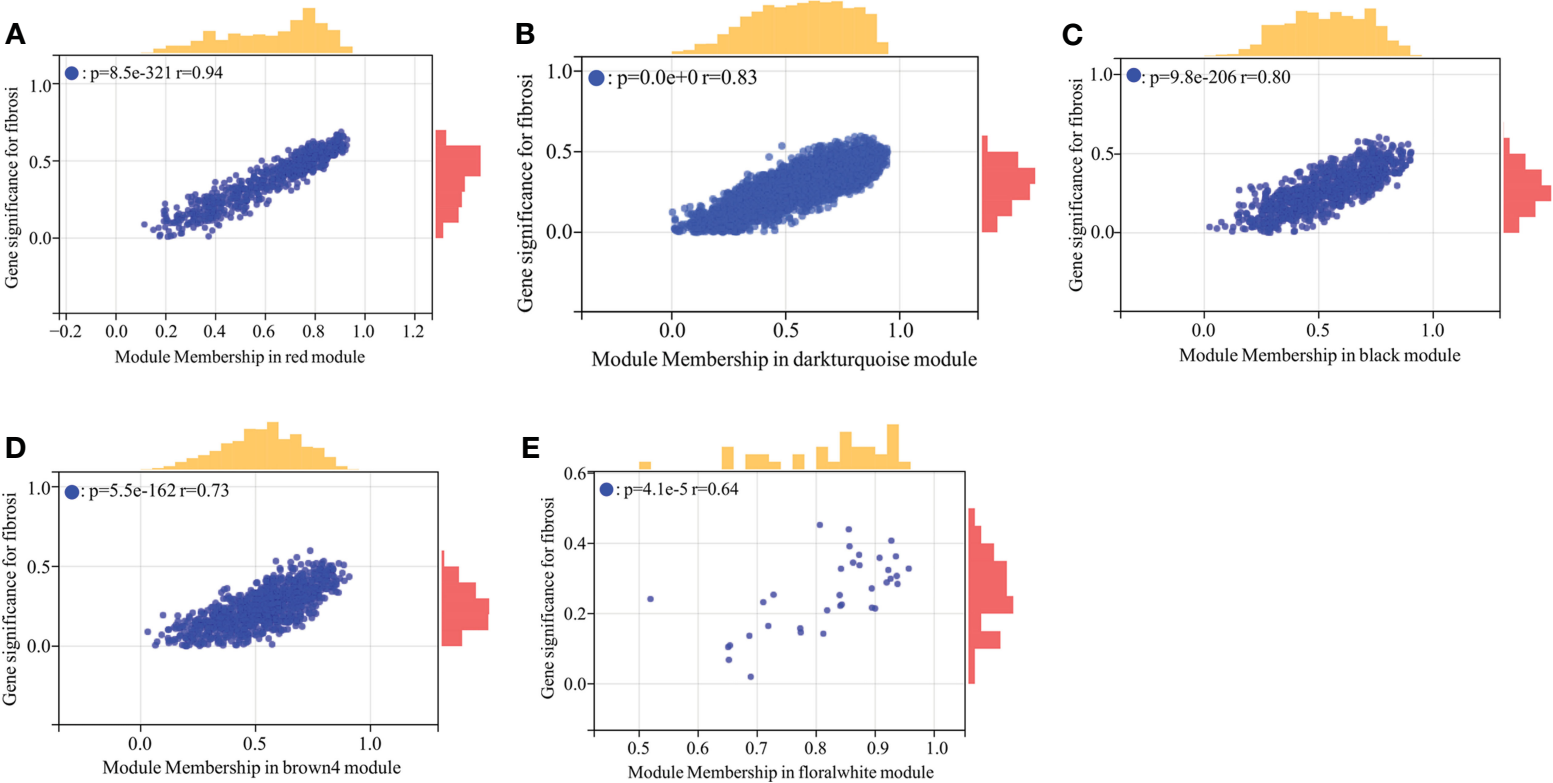
Scatter plot analysis of the modules associated with fibrosis trait. **(A–E)** Scatter plot analysis of the module of red, darkturquoise, black, brown4 and floralwhite, respectively.

The module-trait correlation coefficients were calculated and the results were showed in **Figure 1E**. The results showed that 5 modules including brown4, black, floralwhite, darkturquoise and red were highly relevant with fibrosis and inflammation (pvalue<0.001). Hence, the 5 modules significantly associated with fibrosis and inflammation were selected for further analysis. According to the criteria of |GS|>0.4 and |MM|>0.5 and pvalue<0.05, a total of 684 crucial genes which highly associated with both fibrosis and inflammation were identified from 5 significant modules (**Figures 2**, **3**).

**Figure 4:**
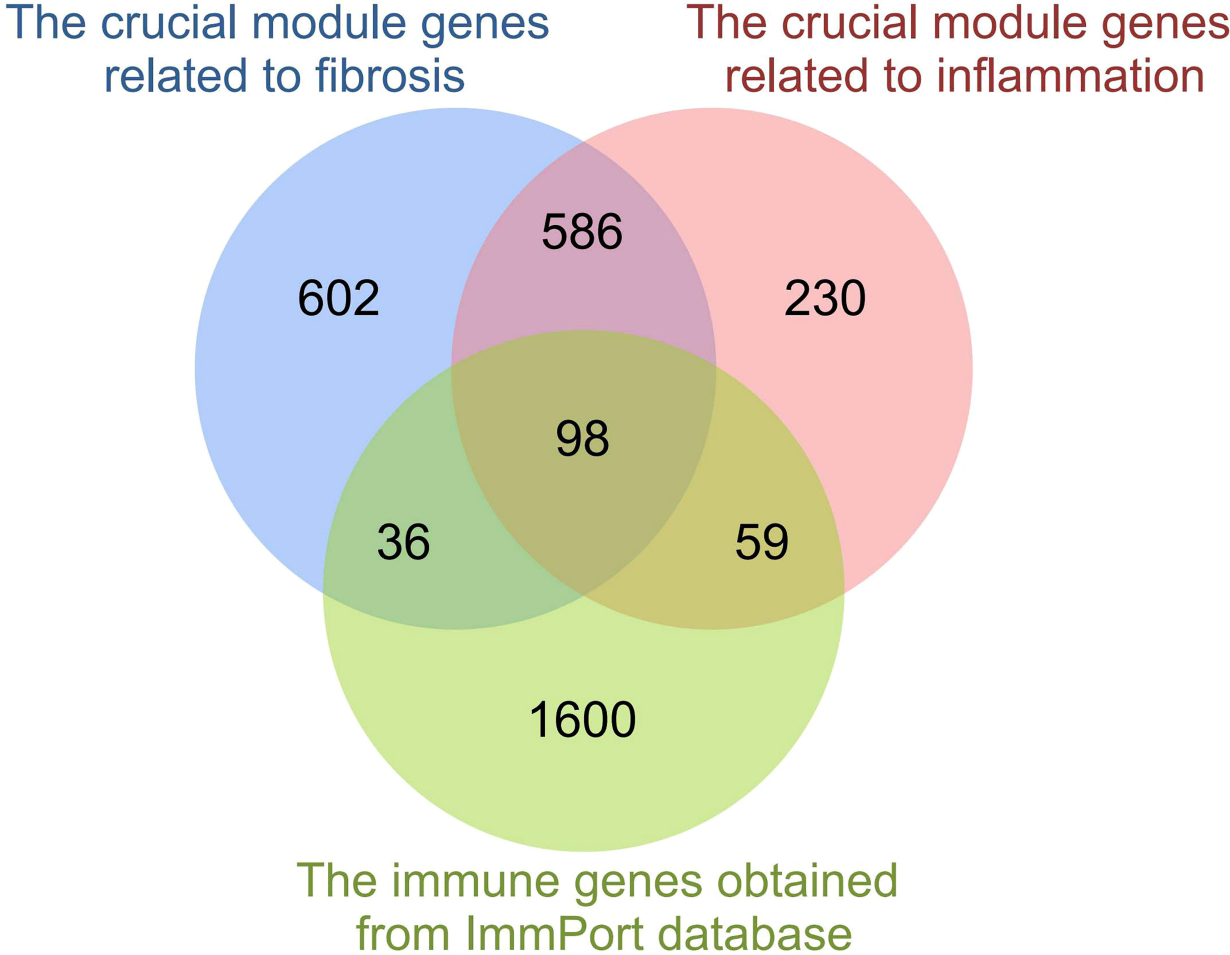
HFIGs identification.

At last, comparing the 2483 immune genes obtained from the ImmPort database, a total of 98 overlapping genes between IRGs and crucial module genes related to fibrosis and inflammation were screened out and identified as hepatic fibrosis immune genes (HFIGs) (**Figure 4**).

### 3.2 Function enrichment analysis of HFIGs

**Figure 5:**
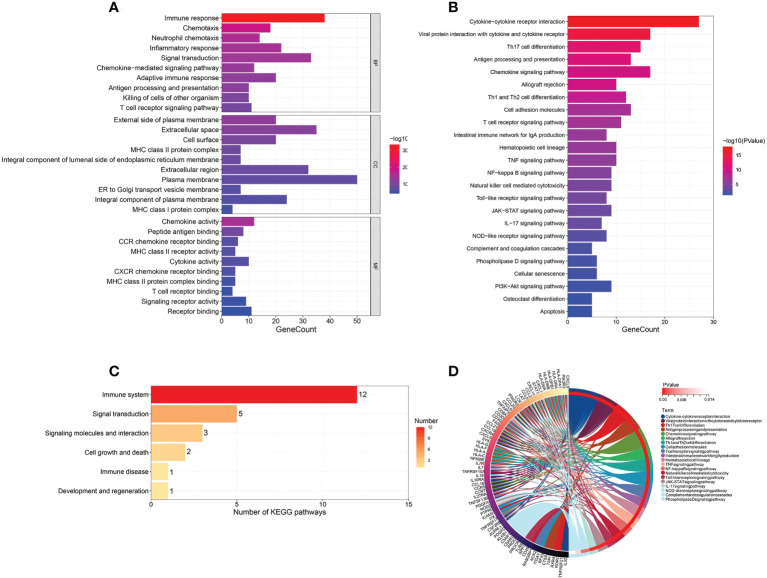
Function enrichment analysis of HFIGs. **(A)** GO annotation of HFIGs. **(B)** KEGG pathway enrichment analysis. **(C)** The classification of KEGG signaling pathways. **(D)** The interaction of KEGG signaling pathways and their associated related HFIGs.

In order to determine the biological functions of HFIGs, 98 HFIGs were uploaded to DAVID Database to perform functional enrichment analysis including GO annotation and KEGG pathway enrichment analysis. GO annotation result showed that the 98 HFIGs were significantly enriched in 149 biological processes (BP), 32 cellular components (CC), and 39 molecular functions (MF), (pvalue<0.05). The BP involved in immune response, chemotaxis, neutrophil chemotaxis, inflammatory response, signal transduction, chemokine-mediated signaling pathway, adaptive immune response, antigen processing and presentationn, killing of cells of other organism, etc. The CC terms included external side of plasma membrane, extracellular space, cell surface, MHC class II protein complex, extracellular region, plasma membrane, ER to Golgi transport vesicle membrane, integral component of plasma membrane and so on. In terms of MF, 98 HFIGs were mostly enriched in chemokine activity, peptide antigen binding, CCR chemokine receptor binding, MHC class II receptor activity, cytokine activity, CXCR chemokine receptor binding, MHC class II protein complex binding, T cell receptor binding and so on. The top 10 most signifcantly enriched GO terms in BP, CC and MF were displayed in **Figure 5A**. In addition, a total of 24 KEGG pathways were obtained after removing the disease related signal pathways (**Figure 5B**). The obtained KEGG pathways involved in cytokine-cytokine receptor interaction, viral protein interaction with cytokine and cytokine receptor, Th17 cell differentiation, antigen processing and presentation, chemokine signaling pathway, TNF signaling pathway, NF-kappa B signaling pathway, Toll-like receptor signaling pathway and so on. Most of the enriched to pathways were immune-related pathways (**Figure 5C**). The connectivity relationships of the HFIGs and their enriched KEGG pathways were displayed in circle chart (**Figure 5D**).

### 3.3 PPI network construction and hub gene selection

**Figure 6:**
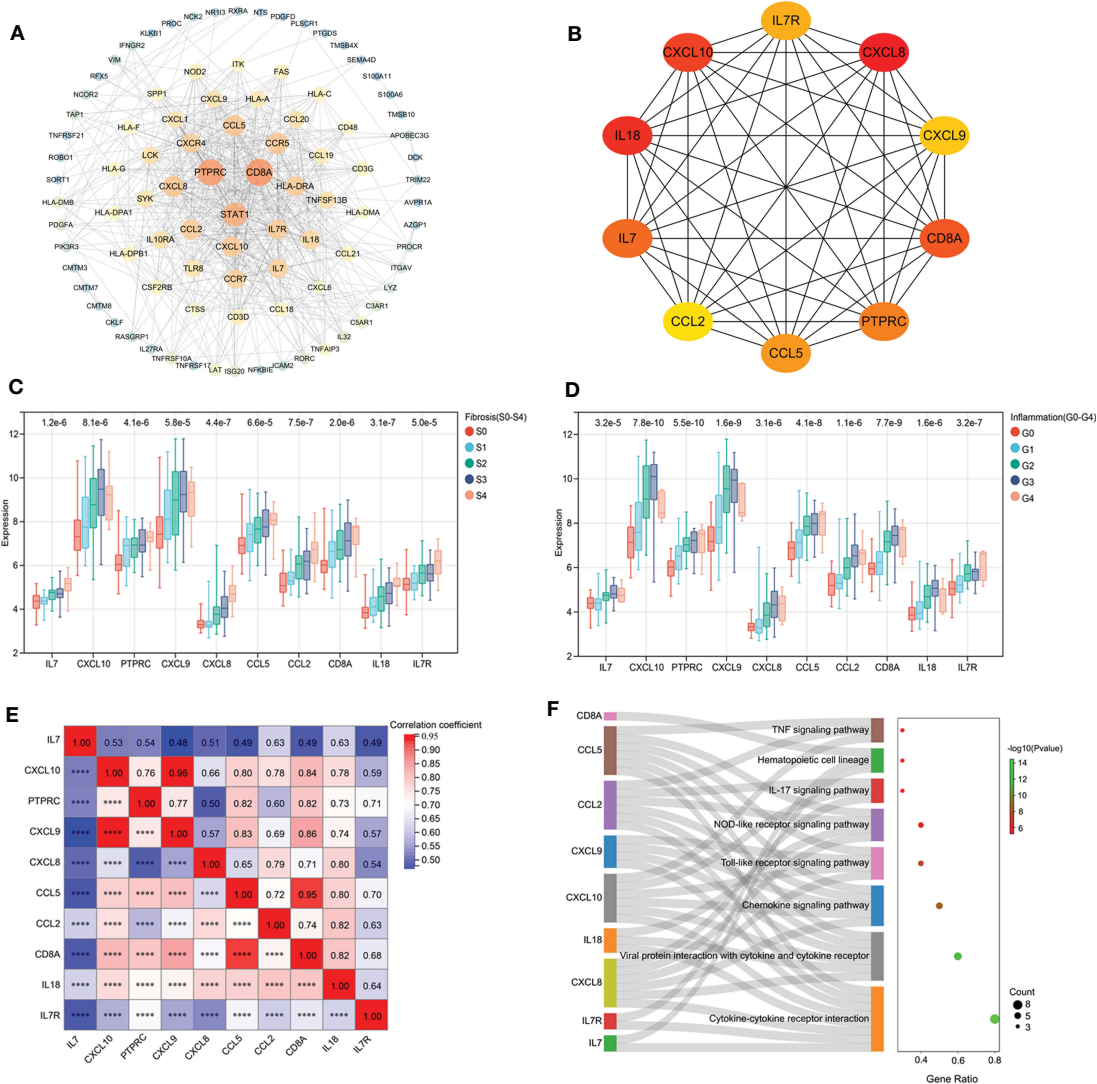
PPI network construction and hub gene selection. **(A)** The PPI network construction. **(B)** 10 hub genes identified from the PPI network. **(C)** The expression of bub genes in liver tissues with different fibrosis stage. **(D)** The expression of bub genes in liver tissues with different inflammatory grade. **(E)** Correlation analysis of hub gene expression. **(F)**: Function enrichment analysis of hub genes.

Using String database and Cytoscape software, the PPI network was constructed (**Figure 6A**). According to MCC algorithm, the 10 top hub gene were screened out from the PPI network and sequentially ordered as follows: CXCL8, IL18, CXCL10, CD8A, IL7, PTPRC, CCL5, IL7R, CXCL9 and CCL2 (**Figure 6B**). The expression of these 10 bub genes was positively correlated with fibrosis stage (S0-S4) and inflammatory grade (G0-G4) (**Figures 6C, D**). All of the hub gene expressions were significantly correlated with each other (pvalue<0.001). High correlations between CXCL9 and CXCL10 (Correlation coefficient=0.96) and between CCL5 and CD8A (Correlation coefficient=0.95) were observed (**Figure 6E**).

Function enrichment analysis showed that the hub genes were enriched in a variety of KEGG pathways. Compared with KEGG pathway enrichment results of 98 HFIGs, 8 common signaling pathways were identified, including cytokine-cytokine receptor interaction, viral protein interaction with cytokine and cytokine receptor, chemokine signaling pathway, Toll-like receptor signaling pathway, NOD-like receptor signaling pathway, IL-17 signaling pathway, hematopoietic cell lineage and TNF signaling pathway (**Figure 6F**).

### 3.4 Immune infiltration analysis

**Figure 7:**
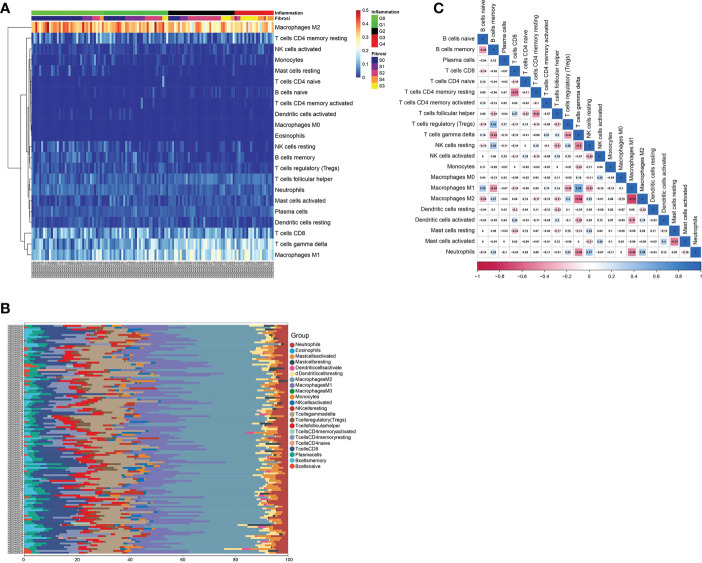
Immune infiltration analysis of GSE84044 data. **(A)** The heat map of immune cell infiltration in the liver tissue of 129 patients with hepatic fibrosis. **(B)** The bar graph of immune cell infiltration in the liver tissue of 129 patients with hepatic fibrosis. **(C)** Correlation between 22 distinct populations of immune cells.

Firstly, we analyzed the infiltration of 22 immune cell types in the liver tissue of 129 patients with hepatic fibrosis. The bar graph and heat map showed the type and number of immune cell infiltrates in each sample (**Figures 7A, B**). 21 infiltrating immune cell types were identified in liver tissue, except for eosinophils, which were not expressed in each sample. Subsequently, we assessed the correlation between these immune cell populations (**Figure 7C**). A positive correlation between macrophages M1 and T cells gamma delta (r=0.56) and a negative correlation between macrophages M2 and macrophages M1 (r=-0.72) and T cells gamma delta (r=-0.58) was found.

**Figure 8:**
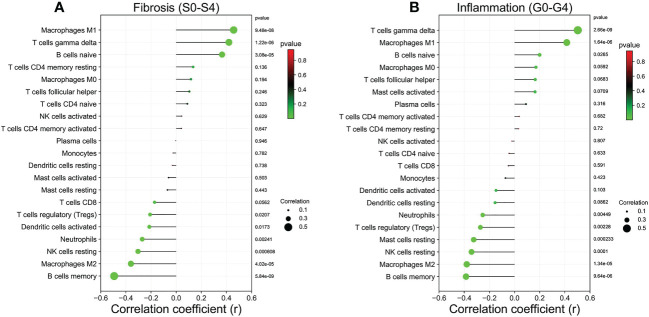
Relationship between the liver tissue fibrosis stage and inflammation grade with immune cell infiltration in patients with HF. **(A)** Liver fibrosis stage (S0-S4). **(B)** Liver inflammation grade (G0-G4).

In addition, the correlation between the infiltration level of 21 immune cells in the liver tissue of each patient and fibrosis stage (S0-S4) and inflammatory grade (G0-G4) was assessed (**Figures 8A, B**). Fibrosis stage (S0-S4) showed a positive correlation with the infiltration status of macrophages M1 (pvalue<0.001), T cells gamma delta (pvalue<0.001) and B cells naive (pvalue<0.001), and a negative link to the infiltration status of B cells memory (pvalue<0.001), macrophages M2 (pvalue<0.001), NK cells resting (pvalue<0.001), neutrophils (pvalue<0.01), dendritic cells activated (pvalue<0.05)and T cells regulatory (Tregs) (pvalue<0.05). Inflammatory grade (G0-G4) was positively correlated with T cells gamma delta (pvalue<0.001), macrophages M1 (pvalue<0.001) and B cells naive (pvalue<0.05), and negatively correlated with B cells memory (pvalue<0.001), macrophages M2 (pvalue<0.001), NK cells resting (pvalue<0.001), mast cells resting (pvalue<0.001), T cells regulatory (Tregs) (pvalue<0.01) and neutrophils (pvalue<0.01).

**Figure 9:**
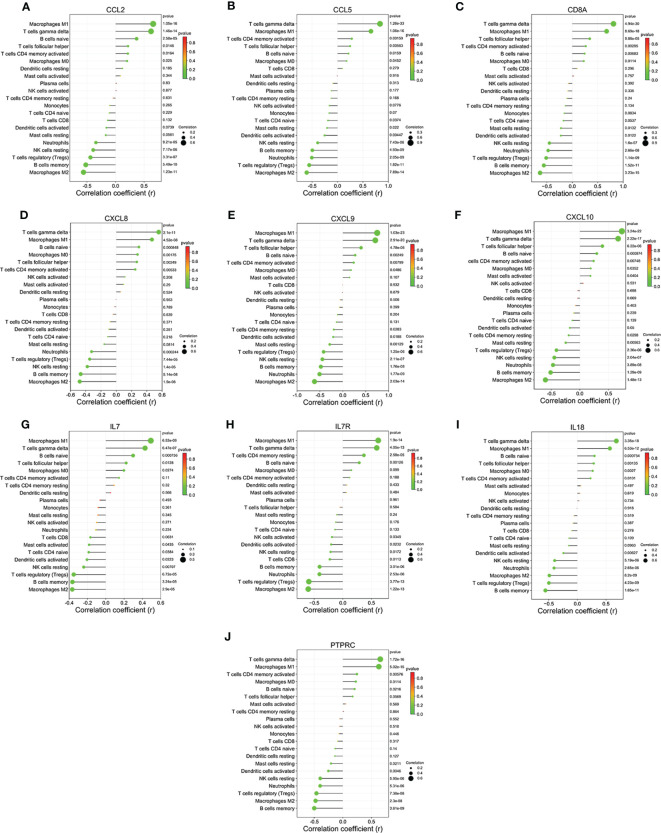
Relationship between hub genes and immune infiltration in liver tissues of HF patients. **(A)** CCL2. **(B)** CCL5. **(C)** CD8A. **(D)** CXCL8. **(E)** CXCL8. **(F)** CXCL8. **(G)** IL7. **(H)** IL7R. **(I)** IL18. **(J)** PTPRC.

Furthermore, We further investigated the correlation of 10 hub genes with 21 immune cell infiltration (**Figures 9A–J**). Each hub gene expression was positively correlated with the infiltration status of macrophages M1, T cells gamma delta and B cells naive, and negatively correlated with the infiltration status of macrophages M2, B cells memory, T cells regulatory (Tregs) and NK cells resting.

### 3.5 Establishment of hepatic fibrosis rat models

**Figure 10:**
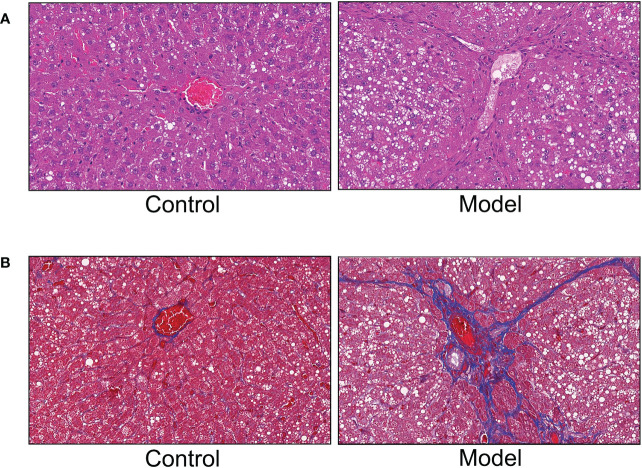
Hematoxylin and eosin (HE) staining and Masson staining. **(A)** HE staining. **(B)** Masson staining.

To verify the reliability of the results of WGCNA analysis, we constructed a rat model of HF. The results of HE and Masson staining were shown in **Figures 10A, B**. The liver tissues in the control group showed a normal structure. However, The liver tissue of model group rats exhibited a large amount of hepatocyte steatosis, pseudolobule formation, hepatocyte swelling and necrosis, infiltration of immune cells and extensive distribution of collagen fibers.

**Figure 11:**
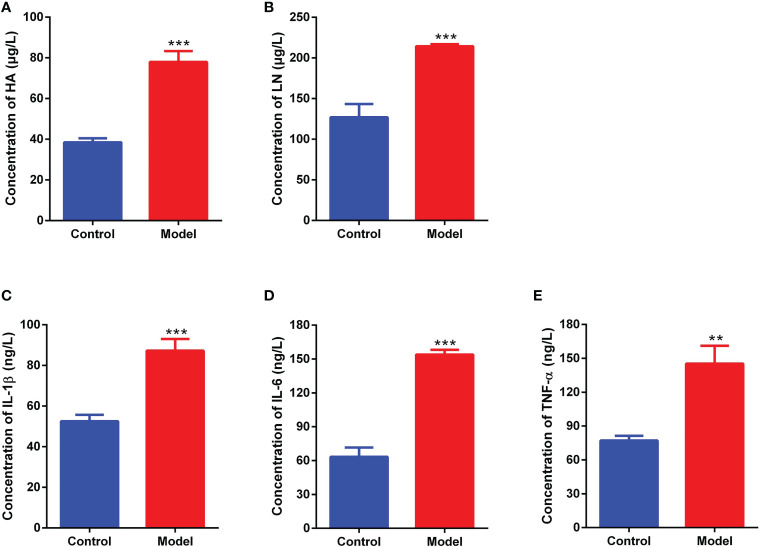
Fibrosis and inflammation parameters of liver tissue measured by ELISA method. **(A)**: Hyaluronic acid (HA). **(B)**: Laminin (LN). **(C)**: Interleukin 1β (IL1β). **(D)**: Interleukin 6 (IL6). **(E)**: Tumor necrosis factor-α (TNF-α). ^**^pvalue <0.01; ^***^pvalue*<*0.001, compared with the normal control group.

Fibrosis and inflammation parameters of liver tissue in the normal control and model groups were measured using ELISA kits. The results were shown in **Figures 11A–E**, compared with the normal control group, The liver collagenic parameters Hyaluronic acid (HA) (pvalue<0.001) and Laminin (LN) (pvalue<0.001) and the liver inflammatory cytokines IL1β (pvalue<0.001), IL6 (pvalue<0.001) and TNF-α (pvalue<0.01) were significantly increased in the CCl4 model group.

### 3.6 Immunofluorescence staining

**Figure 12:**
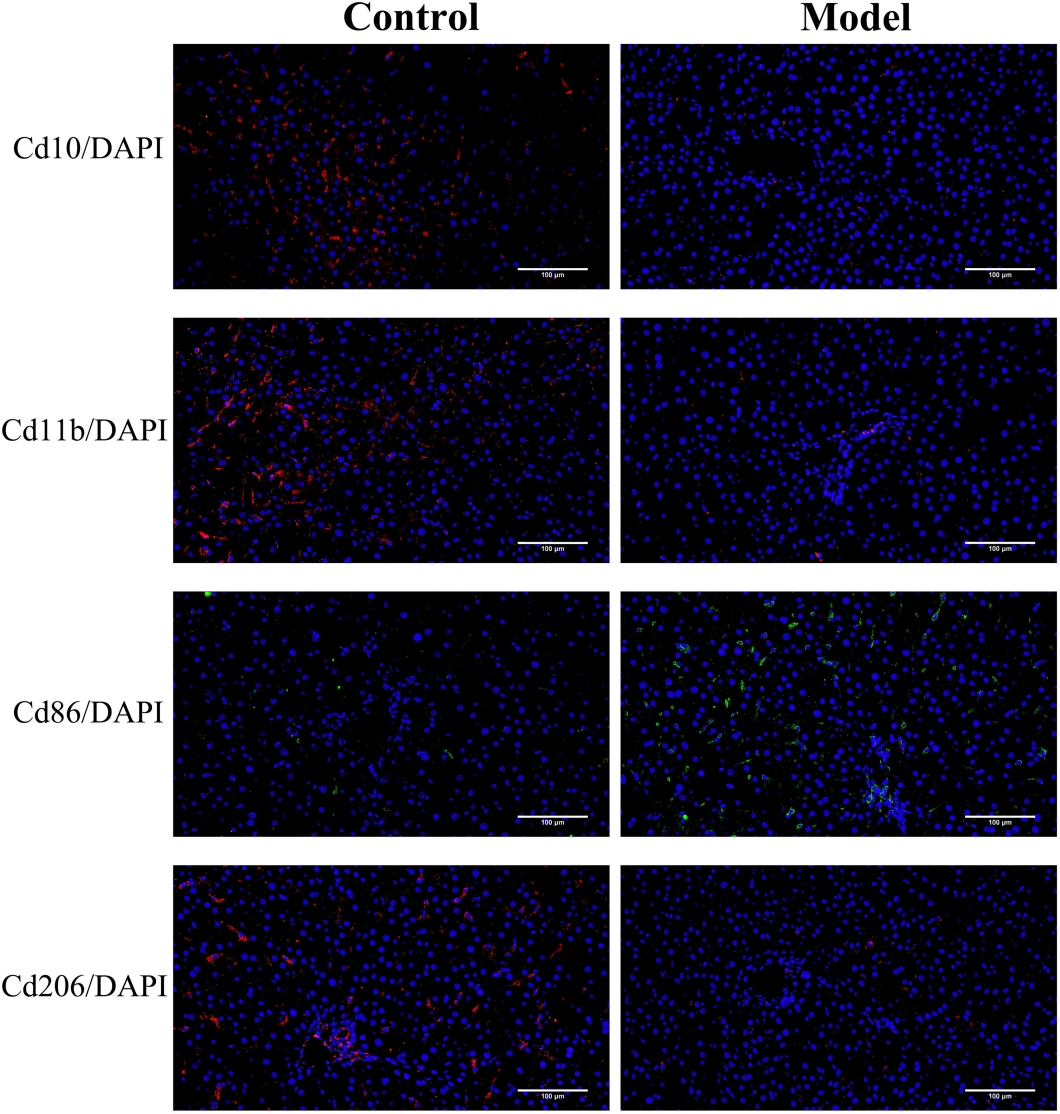
Detection of immune cell infiltration by immunofluorescence staining.

Then, we performed immunofluorescence analysis on the liver tissue of the normal group and the model group. The results were shown in **Figure 12**. The expression level of Cd86 (a marker of macrophages M1) were significantly higher in fibrotic rat liver than that in normal rat liver tissues, but Cd10 (a marker of neutrophils), Cd11b (a marker of NK cells), and Cd206 (a marker of macrophages M2) expression level was significantly decreased in the CCl_4_ model group compared with the normal control group. The results of immunofluorescence staining verified the accuracy of the results of immune infiltration analysis to some extent.

The expression of Cd10, Cd11b, Cd86 and Cd206 were detected to assess the infiltration status of neutrophils, NK cells, macrophages M1 and macrophages M2, respectively.

### 3.7 RNA-seq analysis

**Figure 13:**
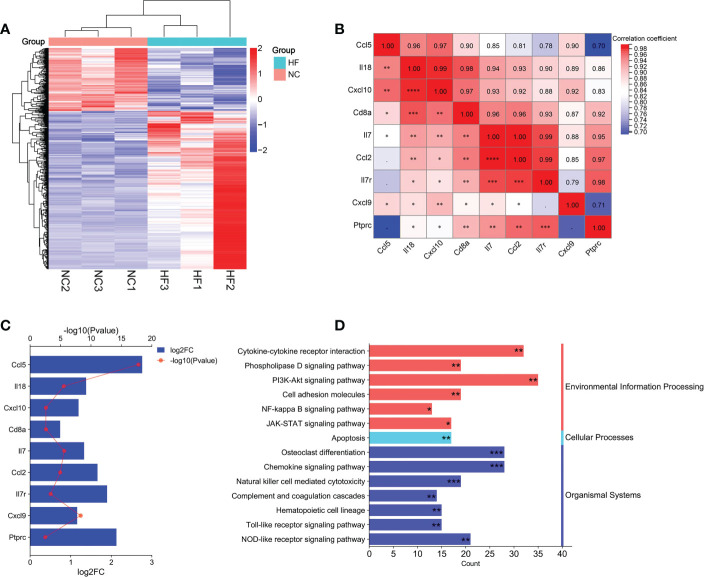
Bioinformatics analysis of RNA-seq data. **(A)** Heatmap of the differentially expressed genes (DEGs) in RNA-seq data. **(B)** Correlation analysis of hub gene expression in RNA-seq data. **(C)** The changes of hub gene expression between the normal and CCl_4_ model groups in RNA-seq data. **(D)**: The common signaling pathways between HFIGs related and DEGs related enriched by DAVID database. ^*^pvalue < 0.05; ^**^pvalue <0.01; ^***^pvalue*<*0.001, indicated the significance of the functional enrichment results.

Liver tissues from normal rats and CCl4-induced HF rats were examined using high-throughput sequencing technology, and then screened for differential genes. Setting |Fold change|>1.5 and pvalue<0.05 as the threshold, 1039 annotated differential genes were screened out, including 744 up-regulated genes and 295 down-regulated genes (**Figure 13A**). Using DAVID database, the 1039 DEGs were enriched in 14 KEGG pathways that were identified as the HFIGs related KEGG signaling pathways in the previous results, including osteoclast differentiation, chemokine signaling pathway, natural killer cell mediated cytotoxicity, complement and coagulation cascades, Toll-like receptor signaling pathway, PI3K-Akt signaling pathway, NOD-like receptor signaling pathway, Apoptosis, NF-kappa B signaling pathway and so on (**Figure 13B**).

Furthermore, we investigated the signaling pathways that were significantly differentially expressed in the model and normal control groups by using GSEA. Compared with 24 pathways that were HFIGs-enriched in the previous results, 19 common KEGG pathways were identified. As shown in **Table 2**, all the common pathways were significantly overexpressed in the model group compared to the normal control group.

**Table 2:** The common signaling pathways between DEGs related enriched by GSEA and HFIGs related enriched by DAVID database.

ID	Description	Enrichment Score	NES	pvalue	qvalue
ko04621	NOD-like receptor signaling pathway	0.52	1.72	1.07E-03	7.94E-03
ko04062	Chemokine signaling pathway	0.58	1.87	1.09E-03	7.94E-03
ko04060	Cytokine-cytokine receptor interaction	0.57	1.83	1.10E-03	7.94E-03
ko04514	Cell adhesion molecules	0.58	1.88	1.10E-03	7.94E-03
ko04380	Osteoclast differentiation	0.65	2.08	1.12E-03	7.94E-03
ko04064	NF-kappa B signaling pathway	0.60	1.87	1.14E-03	7.94E-03
ko04612	Antigen processing and presentation	0.61	1.88	1.14E-03	7.94E-03
ko04650	Natural killer cell mediated cytotoxicity	0.75	2.32	1.15E-03	7.94E-03
ko04658	Th1 and Th2 cell differentiation	0.63	1.91	1.19E-03	7.94E-03
ko04640	Hematopoietic cell lineage	0.73	2.22	1.19E-03	7.94E-03
ko05330	Allograft rejection	0.76	2.16	1.25E-03	7.94E-03
ko04061	Viral protein interaction with cytokine and cytokine receptor	0.69	1.92	1.27E-03	7.94E-03
ko04672	Intestinal immune network for IgA production	0.81	2.15	1.31E-03	7.94E-03
ko04072	Phospholipase D signaling pathway	0.55	1.76	2.20E-03	1.19E-02
ko04620	Toll-like receptor signaling pathway	0.52	1.62	4.55E-03	1.89E-02
ko04659	Th17 cell differentiation	0.50	1.57	6.77E-03	2.50E-02
ko04660	T cell receptor signaling pathway	0.48	1.49	1.26E-02	4.17E-02
ko04151	PI3K-Akt signaling pathway	0.39	1.33	1.64E-02	5.02E-02
ko04610	Complement and coagulation cascades	0.44	1.38	3.64E-02	9.01E-02

9 hub genes were screened from RNA-seq data, including Il18, Cxcl10, Cd8a, IL7, Ptprc, Ccl5, Il7r, Cxcl9 and Ccl2. Cxcl8 did not have any annotation information in rat species and may not be expressed in rat species. The 9 hub genes were significantly upregulated in liver tissue of the CCl4-induced hepatic fibrosis rats compared to the normal liver tissue (**Figure 13C**). Person correlation analysis showed good correlation between all the 9 hub genes (**Figure 13D**). Overall, the results of our transcriptomic analysis further confirm the key roles of these immune-related signaling pathways and hub genes in the progression of HF.

### 3.8 Verification of hub gene expression by q-PCR

**Figure 14:**
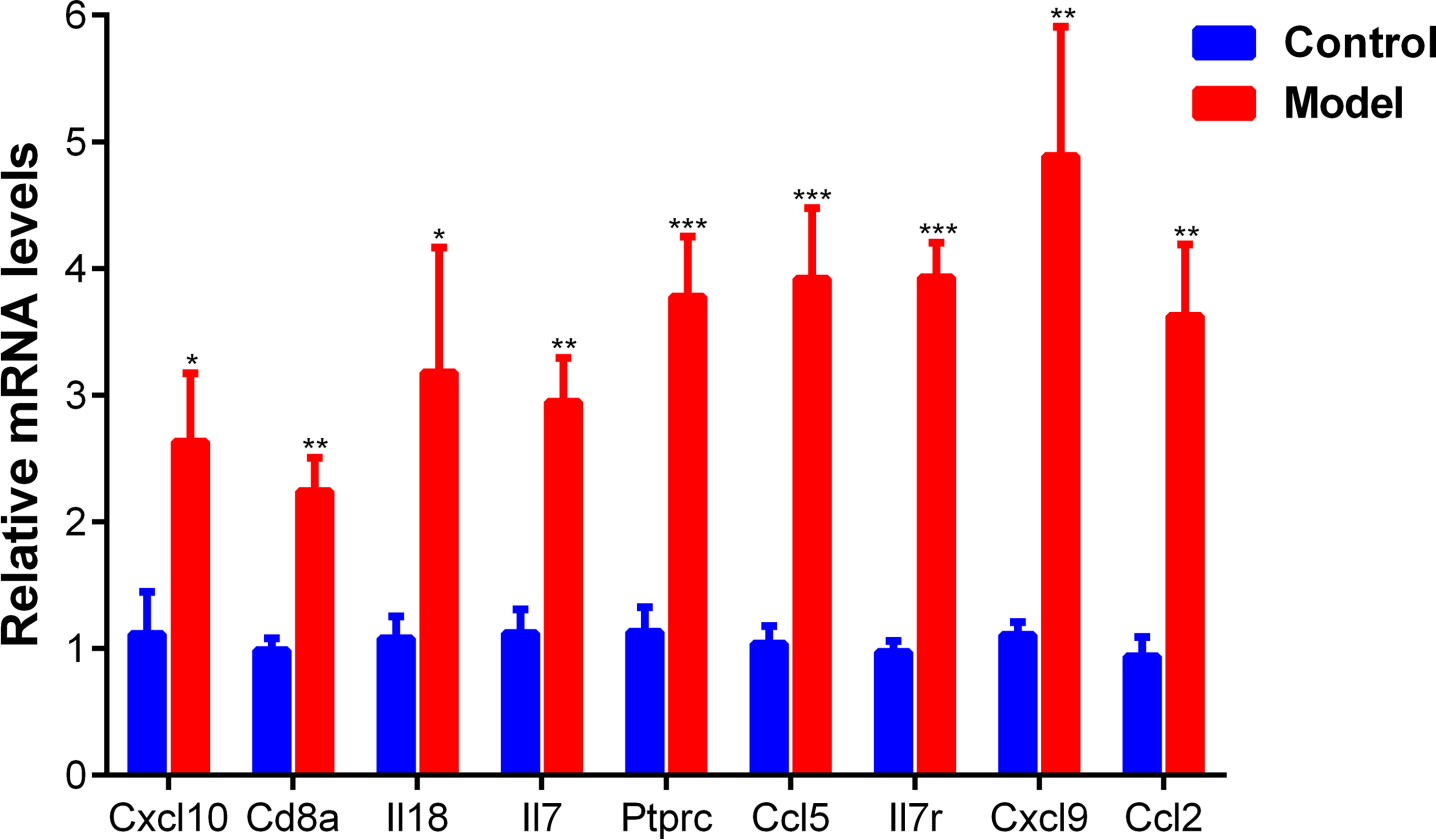
The mRNA expression of 9 hub genes in the normal and CCl4 model groups measured by q-PCR. ^*^pvalue < 0.05; ^**^pvalue <0.01; ^***^pvalue*<*0.001, compared with the normal control group.

Since Cxcl8 is not expressed in rat species, the mRNA expression levels of nine hub genes including Il18, Cxcl10, Cd8a, IL7, Ptprc, Ccl5, Il7r, Cxcl9 and Ccl2 were detected by q-PCR experiments to verify the expression of identified hub genes in liver tissues of the normal group and CCl4 model group. The result was showed in **Figure 14**. The mRNA expression of these 9 hub genes in the CCl4 model group were significantly up-regulated compared to the normal control group, consistent with previous WGCNA analysis and RNA sequencing results (pvalue<0.05, pvalue<0.01, pvalue<0.001).

### 3.9 Identification of candidate small molecules

10 hub genes were uploaded to CMAP to determine potential drugs for reversing HF. The top 15 small molecules with negative connectivity scores were screened out, including telotristat, lomitapide, malotilate, tranylcypromine, TG-101348, cordycepin, tafamidis-meglumine, fostamatinib, delcorine, phenylbutazone, endo-IWR-1, scopolamine, anidulafungin, simvastatin and terconazole (**Table 3**). These small molecule compounds have the potential to exert anti-hepatic fibrosis effects by inhibiting the upregulation of hub genes.

**Table 3:** The small moleculars identification (Top 15).

Rank	CMAP name	Connectivity score	-log (p value)
1	telotristat	-0.912	15.6536
2	lomitapide	-0.883	15.6536
3	malotilate	-0.8735	15.6536
4	tranylcypromine	-0.8728	15.6536
5	TG-101348	-0.8707	15.6536
6	cordycepin	-0.8564	15.6536
7	tafamidis-meglumine	-0.8454	12.2035
8	fostamatinib	-0.8445	11.9793
9	delcorine	-0.8442	2.9231
10	phenylbutazone	-0.8437	2.8391
11	endo-IWR-1	-0.8432	2.7737
12	scopolamine	-0.8392	2.4983
13	anidulafungin	-0.8386	1.9741
14	simvastatin	-0.8371	1.9178
15	terconazole	-0.835	1.8569

## 4 Discussion

Hepatic fibrosis (HF) is the result of an excessive repair response that produces abnormal deposition of extracellular matrix due to liver injury. The inflammatory response due to chronic liver injury is the main pathological factor in the development of HF. Previous studies identified HF biomarkers and their biological functions by bioinformatics analysis. This paper is the first to combine bioinformatics analysis and animal experiments to identify HF-related immune genes and their biological functions and potential regulatory mechanisms. It will help in immune mechanism research and immune diagnosis and therapy of HF.

In this study, 98 HFIGs were first detected in liver tissue of HF patients by WGCNA analysis of GSE84044 data and comparing with the immune genes obtained from the ImmPort database. Subsequently, we carried out functional enrichment analysis of HFIGs and observed that these genes were enriched in 24 signal pathways. Notably, most of the signal pathways were validated in subsequent transcriptomic studies, such as NOD-like receptor signaling pathway, NF-kappa B signaling pathway, Toll-like receptor signaling pathway, PI3K-Akt signaling pathway, T cell receptor signaling pathway and Phospholipase D signaling pathway. Studies have suggested that NOD-like receptor signaling pathway is involved in regulating liver injury and hepatic immune and inflammatory responses (12, 13). NOD-like receptor thermoprotein domain 3 (NLRP3), as a critical target in the NOD-like receptor signaling pathway, contributes to cell death and immune response in HF by forming NLRP3 inflammasome with cysteinyl aspartate specific proteinase-1 (caspase-1) and apoptosis-associated spot-like protein (ASC) (13). NLRP3 inflammasome activates caspase 1 and promotes the maturation and secretion of downstream pro-inflammatory cytokines such as IL-1β and IL-18, resulting in the occurrence of programed cell death (apoptosis, autophagy, pyroptosis), liver inflammation and fibrosis (14–17). The Toll-like receptors (TLRs) belonging to the pattern recognition receptors (PRRs) family are an important component of innate immune defense. TLRs specifically recognize pathogen-associated molecular patterns (PAMPs) and damage-associated molecular patterns (DAMPs), leading to activation of the organism’s immune response. Activation of Toll-like receptor signaling pathway *via* PAMPs or DAMPs is a key factor contributing to the liver immune damage in HF. TLRs including TLR2, TLR3, TLR4 and TLR9 are expressed on hepatocytes, Kupffer cells, fibroblasts, neutrophils, dendritic cells and endothelial cells in the liver and activated in hepatic fibrosis liver tissue. TLR4, a cell surface TLR, is mainly expressed on HSCs, Kupffer cells and hepatocytes in the liver. It recognizes lipopolysaccharides (LPS) and DAMPs, and then activates HSCs and immune signals to secrete large amounts of cytokines and chemokines, resulting in the recruitment and activation of macrophages and hepatocyte death. TLR4 is highly expressed in Kupffer cells of HF and mediates M1-type macrophage polarization *via* TLR4-NF-κB and TLR4-MAPK signaling pathways. Furthermore, previous studies have suggested that TLR2, TLR3, TLR7, TLR8 and TLR9 were also involved in the occurrence and development of HF. X. Xie et al. (18) suggested that TLR2 was the HBeAg receptor and its expression correlates with the degree of inflammation and fibrosis in liver tissue of hepatitis B patients. J. Howell et al. (19) suggested that impaired TLR3 and TLR7/8 function may contribute to liver fiber formation post-liver transplantation with hepatitis C virus (HCV) infection by activating HSCs through the secretion of pro-inflammatory factors. Mice deficient for Tlr9 exhibit reduced HF in the bile duct ligation-induced HF model (20). The PI3K-Akt signaling pathway is activated in HF and the activated PI3K-Akt signaling pathway contributes to increased liver reactive oxygen species, hepatic tissue immune infiltration, hepatocyte apoptosis, hepatic stellate cell activation, and excessive ECM deposition (21–23). More immune-related signaling pathways in HF were identified in this study, and it helps to better investigate the immune mechanisms underlying the development of HF.

Moreover, 10 hub genes including CXCL8, IL18, CXCL10, CD8A, IL7, PTPRC, CCL5, IL7R, CXCL9 and CCL2 were identified from PPI network of 98 HFIGs and the expressions of these hub genes were positively correlated with the degree of liver inflammation and fibrosis. These hub genes were also enriched in the NOD-like receptor signaling pathway, Toll-like receptor signaling pathway and other signaling pathways as HIFGs. Except CXCL8, all hub genes were confirmed to be significantly up-regulated in the liver tissue of CCl4-induced HF rats compared with normal rats in subsequent transcriptome and q-PCR experiments. Moreover, immune infiltration analysis based on GSE84044 revealed that the expression of 10 hub genes was positively correlated with macrophages M1, T cells gamma delta and B cells naive, and negatively correlated with macrophages M2, B cells memory, T cells regulatory (Tregs) and NK cells resting, and that the hub gene-related immune cells are also associated with the fibrosis stage and inflammatory grade of HF. The infiltration status of immune cells in HF was verified in the subsequent immunofluorescence analysis.

Interleukin-8 (CXCL8, IL-8) as a multifunctional pro-inflammatory cytokine, plays an important role in HF and its related inflammatory damage. CXCL8 is highly expressed and secreted in human HF. CXCL8 is released by numerou cell types, including monocytes, macrophages, neutrophils and endothelial cells, and it causes inflammatory damage by recruiting and activating neutrophils and T cells (24, 25). H. Tang et al. (26) found that CXCL8 was highly expressed in serum and liver tissues of patients with chronic liver disease, and its expression was positively correlated with inflammatory cytokines and fibrosis markers in the liver, and CXCL8 recruitted and activated the hepatic macrophages *via* CXCR1 in human non-cholestatic cirrhosis. The CXCL8 expression is induced by a variety of cytokines, such as TNF-α, IL-33, IL-1β and IL-6, and CXCL8 stimulates numerou immune-related signaling pathways, such as Toll-like receptor signaling pathway, NOD-like receptor signaling pathway, MAPK signaling pathway and NF-κB signaling pathway (27–29). Studies showed that CXCL8 promoted hepatic fibrosis by inducing α-smooth muscle actin (α-SMA) expression and stress fiber formation in HSCs (24, 30). B. Langhans et al. (31) suggested that the expression of CXCL8 in liver Tregs was correlated with the stage of fibrosis and the Treg could activate hepatic HSCs *via* CXCL8. The cytokine CXCL8 is a potent interferon gamma inducing factor which mediate T cell proliferation and activation (31). Many liver inflammatory diseases are associated with increased expression of CXCL8. High expression of serum CXCL8 has been observed in patients with chronic liver disease of different etiologies (32). J. Knorr et al. (33) found that the IL-18 expression was elevated in patients with liver cirrhosis compared with healthy controls, and mice deficient for il18 exhibit reduced HF in the nonalcoholic steatohepatitis (NASH)-induced HF model. The hepatic IL-18 was mainly expressed in Kupffer cells and IL-18 promoted HSC activation and remarkable collagen deposition by activating NLRP3 inflammasome (33). C-X-C motif chemokine 10 (CXCL10) promotes liver inflammation in chronic or acute liver injury by recruiting and activating immune cells such as B-lymphocytes, T-lymphocytes and dendritic cells (34). E. Hintermann et al. (34) found that CXCL10 acts as a pro-fibrotic factor by regulating hepatocytes, natural killer cells and HSCs. C-X-C motif chemokine 5 (CCL5) is an inflammatory cytokines that is strongly expressed in HF. CCL5 was initially detected as a T-cell-specific molecule, but has since been found to expressed in natural killer cells, HSCs, endothelial cells and Kupffer cells. The CCL5 activates and recruits immune cells such as NK cells, macrophages, T cells and B cells to sites of liver inflammation by interacting with its specific receptors in the hepatic fibrosis process (35, 36). M.L. Berres et al. (36) found that antagonism of the chemokine Ccl5 could improve fibrosis and inflammatory damage in the CCl4-induced HF mices and methionine and choline-deficient (MCD) diet-induced HF mices. Kupffer cell and T cells activation and infiltration contribute to the occurrence of steatosis, fibrosis formation and inflammatory injury in alcoholic liver disease (ALD). A. Ambade et al. (37) showed that CCL2 and CCL5 were highly expressed in the ALD, and inhibition of CCR2/5 signaling could improve fibrosis, steatosis, and inflammatory damage in the mouse model of ALD by reducing macrophage infiltration and inhibiting inflammatory factor secretion. Taken together, these HFIGs especially the 10 hub genes CXCL8, IL18, CXCL10, CD8A, IL7, PTPRC, CCL5, IL7R, CXCL9 and CCL2 may play a key role in HF and its inflammatory damage.

To explore immune target-based therapeutics for HF, we used the CMAP platform to screen 15 small molecule compounds with the potential to reverse the high expression of hub genes, including telotristat, lomitapide, malotilate, tranylcypromine, TG-101348, cordycepin, tafamidis-meglumine, fostamatinib, delcorine, phenylbutazone, endo-IWR-1, scopolamine, anidulafungin, simvastatin and terconazole. Notably, some of these small molecules have been reported for their anti-liver fibrosis effects, like malotilate (38), TG-101348 (39), cordycepin (40, 41), simvastatin (42, 43). Tranylcypromine is a targeted inhibitor of monoamine oxidase (MAO) (44), which plays an important role in the development of HF as a marker for the clinical diagnosis of HF (45, 46). It suggests that tranylcypromine may reverse the development of HF by inhibiting MAO expression. However, the effects and mechanisms of other small molecule drugs on HF are still unclear, more *in vivo* and *in vitro* experiments are needed for verification.

## 5 Conclusion

In conclusion, this study demonstrated that immune mechanism play a key role in the occurrence and development of HF, and explored immune target-based therapeutics for HF. We screened 98 immune genes associated with HF and identified 10 hub genes from them, including CXCL8, IL18, CXCL10, CD8A, IL7, PTPRC, CCL5, IL7R, CXCL9 and CCL2. We detected the 9 hub genes expression in liver tissue of HF rats and the results suggested that these hub genes may contribute to the fibrosis formation and inflammatory damage in HF. We identified several immune signaling pathways associated with HF, such as NOD-like receptor signaling pathway, NF-kappa B signaling pathway, Toll-like receptor signaling pathway, PI3K-Akt signaling pathway, T cell receptor signaling pathway and Phospholipase D signaling pathway. Immune infiltration analysis and immunofluorescence analysis suggested that the infiltration status of immune cells like neutrophils, NK cells, macrophages M1 and macrophages M2, were significantly correlated with HF. Moreover, Collectively, this study revealed the potential immune mechanism of HF and investigated immune therapeutic agents to reverse HF, the specific molecular mechanisms of the disease and the pharmacological mechanisms of small molecule drugs need to be further explored.

## Data availability statement

The datasets presented in this study can be found in online repositories. The names of the repository/repositories and accession number(s) can be found below: PRJNA925303 (Bioproject).

## Ethics statement

The animal study was reviewed and approved by the Ethical Committee of Ningxia Medical University.

## Author contributions

The authors’ contributions are as follows: Experiments and data analysis: Y-MB. He is the first author. Grammar modification: SL and BZ. All authors contributed to the article and approved the submitted version.
